# Comprehensive analysis of cellular senescence and immune microenvironment in papillary thyroid carcinoma

**DOI:** 10.18632/aging.205520

**Published:** 2024-02-07

**Authors:** Yinde Huang, Han Jiang, Guangwen Xu, Xin Li, Wenbin Chen, Yu Lun, Jian Zhang

**Affiliations:** 1 Department of Vascular and Thyroid Surgery, The First Affiliated Hospital of China Medical University, Shen-Yang 110001, Liaoning, China; 2 Department of Breast and Thyroid Surgery, Chongqing General Hospital, Chongqing 401147, China

**Keywords:** papillary thyroid cancer, cellular senescence, aging, overall survival, TCGA

## Abstract

Senescence-induced therapy was previously considered as an effective treatment for tumors, and cellular senescence was initially regarded as an effective mechanism against cancer. However, whether cell senescence-related genes can be used to predict the prognosis of papillary thyroid carcinoma (PTC) and immunotherapy remains unclear. We developed and validated a cell senescence-related signature (CSRS) by analyzing the gene expression of 278 genes related to cellular senescence in 738 patients with PTC. Additionally, further analysis showed that CSRS was a reliable predictor of patient outcomes in combination with immune checkpoint expression and drug susceptibility, and patients with high risk scores may benefit from immunotherapy. The findings of this study demonstrate that CSRS serves as an immunotherapeutic response and prognosis biomarker affecting the tumor immune microenvironment of PTC.

## INTRODUCTION

The rapid increase in the incidence of thyroid cancer (TC) has attracted worldwide attention. It is estimated that there will be 224,023 new cases in China [1] and 43,800 new cases in the United States in 2022 [2]. The widespread use of high-resolution imaging techniques such as ultrasonography has led to a sharp increase in the incidence of papillary thyroid carcinoma (PTC) [3–6]. The primary treatment option for PTC patients is surgical, with thyroid hormone supplementation required in all thyroidectomy and more than two-thirds of lobectomies [7, 8]. Given that some PTC cases display indolent behavior, surgeons have started considering active surveillance as an alternative to extensive surgery to reduce overtreatment and healthcare costs [9]. Further research on the pathogenesis and prognostic characteristics of PTC is needed to identify high-risk PTC patients and facilitate targeted treatment.

The 2017, the American Joint Committee on Cancer (AJCC) emphasized the significance of age in TNM staging of thyroid cancer [10]. From 1990 to 2017, the age-standardized incidence of TC in both China and the United States showed an upward trend [11]. Therefore, it is particularly important to elucidate the pathogenesis and prognostic value of aging in PTC.

Aging is defined as a decline in physiological function over time, leading to an increased likelihood of disease [12]. One of the major features of aged organs is the accumulation of cellular senescence [13–15]. Cellular senescence is originally defined as cells reaching their replication limit and being permanently arrested in their cell cycle [16]. Among the cancer hallmarks suggested for the third edition in 2022, four new ones were added, including senescent cells [17]. Targeting the activation or inhibition of cellular senescence is beneficial for tumor immunotherapy [18]. Although several lines of evidence indicate that young adults with cancers demonstrate unique histological and survival heterogeneity, their biology is incompletely understood [19, 20]. In our current study, we analyzed 42,756 patients with PTC in the Surveillance, Epidemiology, and End Results (SEER) database and found that overall survival (OS), disease-specific survival (DSS), progression-free survival (PFS) were shorter in those aged 55 years or older. To comprehensively assess the role of cellular senescence in PTC progression and the potential to screen high-risk patients, we performed cluster analysis on PTC patients using cellular senescence-related genes and constructed a cell senescence-related signature (CSRS) from the Genotype-Tissue Expression (GTEx), the Cancer Genome Atlas - Thyroid Carcinoma (TCGA – THCA) and eight cohorts in the Gene Expression Omnibus (GEO). Consensus clustering found that the overall OS of cluster 2 population was worse. Subsequently, we constructed CSRS through four genes (SNAI1, CDKN2A, HDAC4, NDRG1), and found that the degree of immune cell infiltration, clinicopathological characteristics of high-risk groups were significantly different, and the OS was worse. Finally, the CSRS can be used to predict the response of PTC patients to immunotherapy and chemotherapy drugs.

## MATERIALS AND METHODS

### SEER cohort, TCGA - THCA cohort, GTEx and GEO cohorts

**Figure 1:**
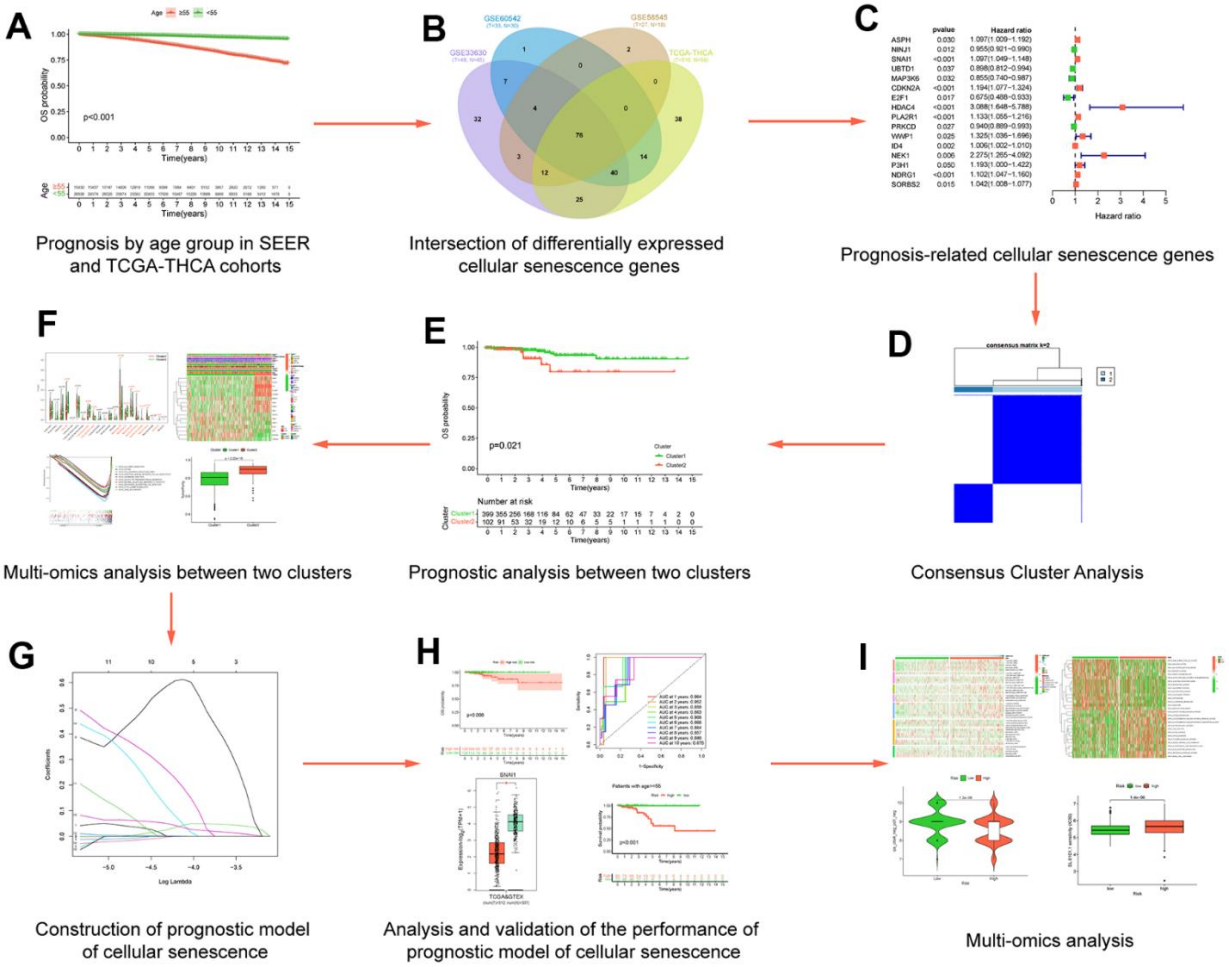
**Flow chart of data analysis.** (**A**) Prognosis by age group in SEERand TCGA-THCA cohorts; (**B**) Intersection of differentially expressed cellular senescence genes; (**C**) Prognosis-related cellular senescence genes; (**D**) Multi-omics analysis between two clusters; (**E**) Prognostic analysis between two clusters; (**F**) Consensus Cluster Analysis; (**G**) Construction of prognostic model of cellular senescence; (**H**) Analysis and validation of the performance of prognostic model of cellular senescence; (**I**) Multi-omics analysis.

We followed the flowchart outlined in Figure 1 for our analysis. The SEER database is an authoritative cancer statistical database in the United States, recording the incidence, mortality, and morbidity of millions of patients with malignant tumors in various states and counties. Our study included patients diagnosed with PTC as their first primary tumor in the SEER cohort between 2004 and 2015. The TCGA-THCA dataset provides comprehensive information, including transcriptome profiling, copy number variation, DNA methylation, and somatic structural variation for a large number of cancer patients. We included PTC cases from the TCGA-THCA cohort with complete follow-up information, and we retrieved clinical data from the University of California at Santa Cruz (UCSC) Xena website (https://xena.ucsc.edu/). Transcriptome data of PTC tissues and normal tissues were obtained from the TCGA (T=510, N=58), GTEx (N = 337) and eight GEO cohorts (http://www.ncbi.nlm.nih.gov/geo): GSE33630 (T=49, N=45), GSE60542 (T=33, N=30), GSE58545 (T=27, N=18), GSE3467 (T=9, N=9), GSE3678 (T=7, N=7), GSE5364 (T=35, N=16), GSE27155 (T=51, N=4), GSE53157 (T=15, N=3). Since we obtained the data from public source websites, no ethical review was required.

### Consensus clustering analysis based on genes related to cellular senescence

We extracted 278 genes related to cellular senescence from the CellAge website [21] (https://genomics.senescence.info/cells/) (Supplementary Table 1), which contains manually curated gene information related to cellular senescence. We analyzed the cell senescence genes extracted from PTC and normal tissues in TCGA, GSE33630, GSE60542 and GSE58545 through the “limma” package [22] of R software (R 4.1.0) to obtain differentially expressed genes (DEG) (FDR < 0.05), and then took the intersection [23]. The TCGA-THCA and GTEx data were batch corrected using the “normalizeBetweenArrays” function of the “limma” package. In cases where probe data shared the same gene name, we aggregated them to calculate the average expression. Subsequently, we performed Gene Ontology (GO) and Kyoto Encyclopedia of Genes and Genomes (KEGG) functional enrichment analyses on the genes in the intersection. We also constructed a protein-protein interaction (PPI) network using the STRING website (https://string-db.org/) and Cytoscape software (version 3.8.2). Screening for prognostic genes associated with cellular senescence was done with a Cox analysis. Univariate Cox analysis was used to screen cellular senescence genes associated with OS (*p* < 0.05) and the expression levels of these genes were displayed with a heatmap.

In accordance with the expression of OS-related cellular senescence genes, we conducted a consensus cluster analysis on PTC patients and examined Kaplan-Meier (K-M) curves, clinicopathological characteristics, immune cell infiltration, immune microenvironment, and KEGG enrichment pathways among different clusters.

The abundance of immune cell infiltration was assessed using the CIBERSORT algorithm [24, 25], which can determine the abundance of 22 types of immune cells in the tissue. The tumor microenvironment was evaluated using four indicators (ESTIMATEScore, StromalScore, ImmuneScore and TumorPurity) using the ESTIMATE algorithm [26].

### Establishing and validating a prognostic model

To quantitatively analyze the impact of cellular senescence genes on the prognosis of PTC patients, we randomly divided the TCGA-THCA cohort into a training cohort and a validation cohort (1:1). We constructed a CSRS by performing Least absolute shrinkage and selection operator (LASSO) regression analysis [27, 28] on the prognosis-related cellular senescence genes in the training cohort using the “glmnet” package [29]. Based on the prognostic model, we performed risk score calculations for patients in the training cohort, validation cohort, and the entire TCGA-THCA cohort. The calculation formula of the CSRS was as follows [28]:


$$CSRS=\sum_{i=1}^{n}Exp_{i}*Coef_{i}$$

where *Exp_i_* is the expression value of the i gene in the model, and *Coef_i_* is the coefficient calculated by LASSO.

We conducted CSRS (Cellular Senescence Risk Score) calculations for patients within the training cohort, validation cohort, and the entire TCGA-THCA cohort. Based on the median risk scores, patients were stratified into high-risk and low-risk groups.

Subsequently, we performed a comprehensive array of analyses, including K-M survival curves, receiver operating characteristic (ROC) curves, decision analysis curves, survival status maps, and heatmaps illustrating the genes associated with CSRS in distinct patient groups.

The expression levels of genes involved in the construction of the CSRS were verified by the GEPIA2 [30] website (http://gepia2.cancer-pku.cn/#index) and in five GEO datasets: GSE3467 (T=9, N=9), GSE3678 (T=7, N=7), GSE5364 (T=35, N=16), GSE27155 (T=51, N=4), GSE53157 (T=15, N=3).

### Hierarchical analysis

To further mitigate the potential influence of clinicopathological features on CSRS as a prognostic factor, we conducted a stratified prognostic analysis based on various clinicopathological features within the entire TCGA-THCA cohort. These features include age [≥55 vs. < 55 years], gender [male vs. female], AJCC stage [III - IV vs. I - II], M [M1 vs. M0], N [N1 vs. N0], T [T3 - 4 vs. T1 - 2], BRAF [mutated vs. wild-type], RAS [mutated vs. wild-type], Cell Type [Classical vs. Follicular], Radiation therapy [Yes vs. No].

### Multi-omics analysis

In the entire TCGA-THCA cohort, using seven algorithms (TIMER [31], CIBERSORT [24, 25], CIBERSORT-ABS [24], QUANTISEQ [32], MCPCOUNTER [33], XCELL [34], EPIC [35]), and the analyzed algorithms will be employed to elucidate the extent of immune cell infiltration among different groups and identify immune cells playing pivotal roles. We created immune cell heatmaps to compare infiltration rates between high- and low-risk groups.

To further analyze the differences in pathway enrichment between high and low risk groups, we performed Gene Set Variation Analysis (GSVA) with the “GSVA” package [36]. The gene set of “c2.cp.kegg.v7.4.symbols.gmt” was obtained from the MSigDB website [37], and statistics were considered significant was defined as p-values less than 0.05.

Subsequently, we employed clinical correlation heatmaps to illustrate the distribution of clinicopathological features in the high and low-risk groups, along with the expression of genes involved in the CSRS.

The immunophenoscore (IPS) serves as a valuable indicator for predicting the efficacy of anti-CTLA-4 and anti-PD-1 immunotherapy in tumor patients [25]. We obtained the IPS score of PTC patients from The Cancer Immunome Atlas (https://tcia.at/) and used the violin plot to display differences in IPS scores between high and low risk groups.

A model for tumor immune evasion, known as Tumor Immune Dysfunction and Exclusion (TIDE: http://tide.dfci.harvard.edu/) can offer insights into the effects of immunotherapy on two primary mechanisms [38, 39]. We uploaded the gene matrix from TCGA-THCA to the TIDE website and generated violin plots depicting TIDE scores, Dysfunction scores, Exclusion scores and CAF scores between high and low risk groups.

Some advanced cancers have been treated better with immune checkpoint inhibitors (ICIs) [40]. Boxplots, based on 47 immune checkpoints obtained from the literature [41, 42], were used to illustrate the differential expression of immune checkpoints in high-risk and low-risk groups.

Based on information from the Genomics of Drug Sensitivity in Cancer (GDSC) database, an assessment of PTC patients’ response to chemotherapeutic and targeted treatment agents was conducted. The half maximal inhibitory concentration (IC50) of pharmacological agents was estimated with the “pRRophetic” R package, which has been extensively used in medical research [43].

### Data availability statement

All the data come from public databases.

## RESULTS

### Age is a prognostic indicator in SEER and TCGA-THCA cohorts

**Figure 2:**
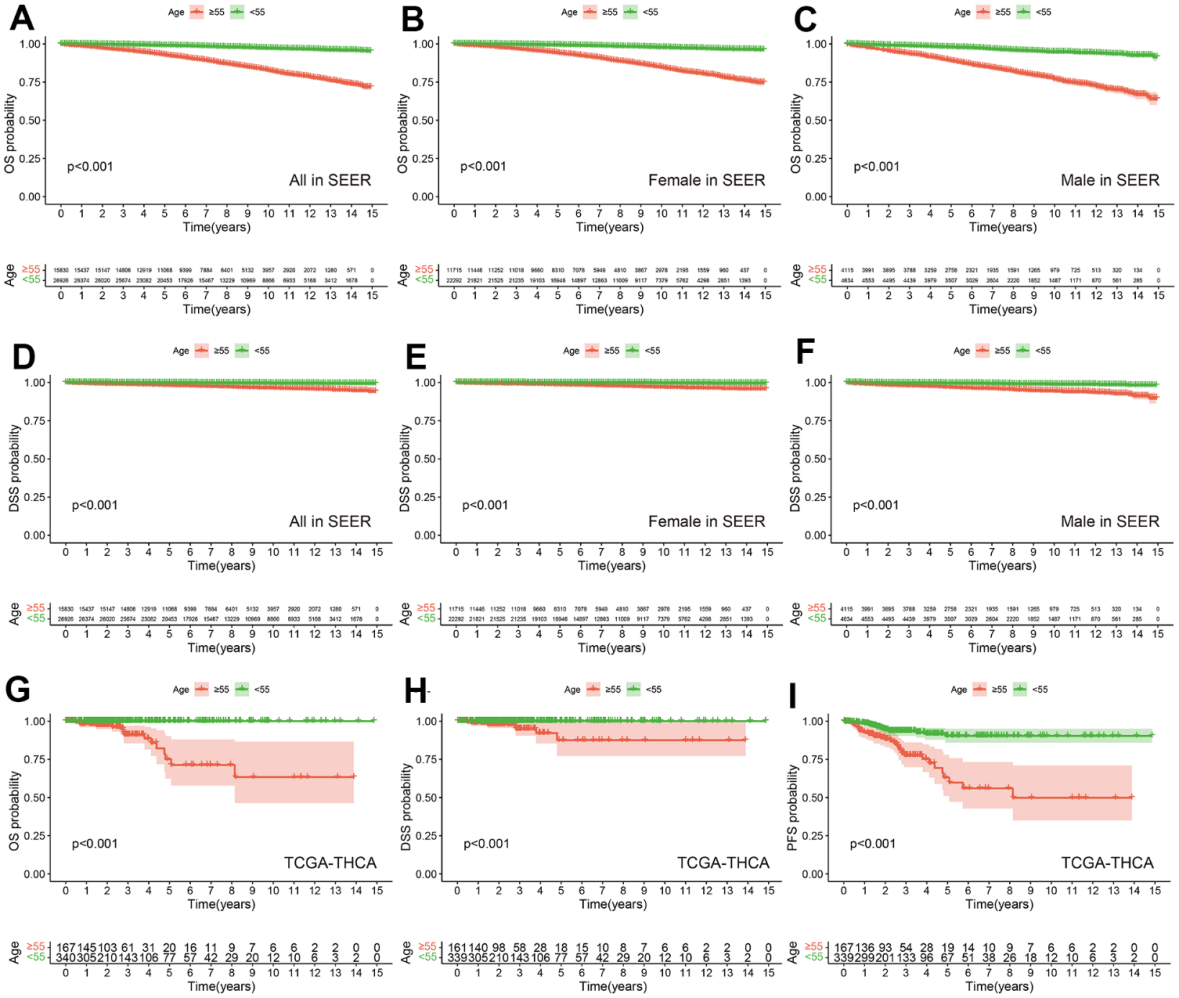
**Prognostic differences among different age groups in SEER and TCGA-THCA cohorts.** OS between PTC with Age ≥55 years and Age <55 years in all (**A**), female (**B**) and male (**C**) in SEER. DSS between PTC with Age ≥55 years and Age <55 years in all (**D**), female (**E**) and male (**F**) in SEER. OS (**G**), DSS (**H**) and PFS (**I**) between PTC with Age ≥55 years and Age <55 years in TCGA-THCA.

According to inclusion and exclusion criteria, we included a total of 42,756 patients in the SEER database. We divided patients into two groups based on age (<55 years, ≥55 years). When OS was the outcome event, patients aged 55 or older also had a worse prognosis than the younger group (Figure 2A). Considering the differences in incidence and mortality of thyroid cancer in different gender populations [44–46], we presented the OS data for the younger and older age groups stratified by gender. The results showed that there was a statistically significant difference in overall survival between the young and old groups of women and men (Figure 2B, 2C). Similar results were obtained when we used DSS as the outcome event (Figure 2D–2F). In the TCGA-THCA cohort, when we used OS, DSS, and PFS as outcome events, the older group also had a worse prognosis than the younger group (Figure 2G–2I).

### The cluster with shorter overall survival could be distinguished based on cell senescence genes

**Figure 3:**
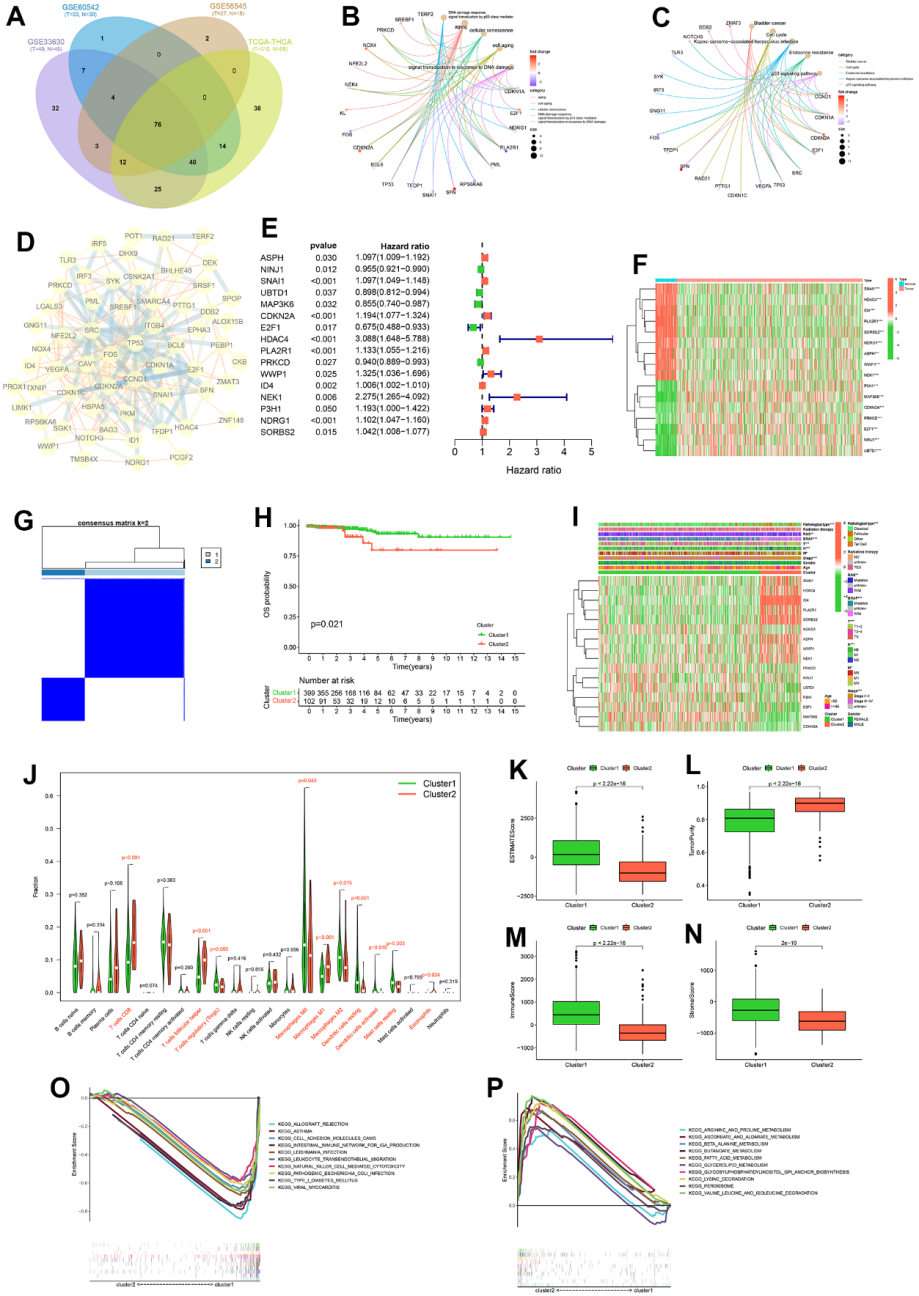
**Consensus cluster analysis.** Intersection of differentially expressed cellular senescence genes across four cohorts (**A**). GO functional enrichment analysis (**B**) and KEGG pathway enrichment analysis (**C**) of 76 intersecting genes. Protein-Protein interactions (PPI) network diagram of 76 intersecting genes (**D**). Forest plot of prognostic genes among 76 intersecting genes (**E**). Expression heatmap of prognostic genes (**F**). Consensus clustering matrix for k = 2 (**G**). K-M curve of OS probability between cluster1 and cluster2 (**H**). Heatmap of clinicopathological feature correlations between cluster1 and cluster2 (**I**). Violin plots of the infiltration of immune cells in cluster1 and cluster2 by CIBERSORT algorithm (**J**). Boxplot of ESTIMATEScore (**K**), TumorPurity (**L**), ImmuneScore (**M**) and StromalScore (**N**) on cluster1 and cluster2 by ESTIMATE algorithm. KEGG pathway enrichment analysis in cluster1 (**O**) and cluster2 (**P**).

278 cellular senescence genes expression were differentially analyzed between tumors and normal tissues in the TCGA-THCA, GSE58545, GSE60542 and GSE33630 cohorts. This analysis identified 76 genes that were common across these datasets (Figure 3A and Supplementary Tables 2, 3). GO functional enrichment analysis showed that 76 intersecting genes were mainly enriched in aging; cell aging; cellular senescence; DNA damage response, signal transduction by p53 class mediator; signal transduction in response to DNA damage (Figure 3B). GO functional enrichment analysis indicated that these genes were mainly involved in aging-related functions. KEGG pathway enrichment analysis showed that these genes were enriched in Bladder cancer, Cell cycle, Endocrine resistance, Kaposi sarcoma - associated herpesvirus infection, p53 signaling pathway (Figure 3C). The PPI network diagram of the proteins corresponding to the 76 genes was shown in Figure 3D. It should be noted that P53 protein has many protein interactions, and both GO function enrichment analysis and KEGG pathway enrichment analysis involved p53 signaling mediated regulatory signals. It may be that P53 is involved in cell senescence process in PTC.

We performed univariate Cox analysis on the OS of PTC patients based on the expression of these 76 genes, and obtained 16 prognosis-related cellular senescence genes (Figure 3E and Supplementary Figure 2). The expression levels of these 16 genes (ASPH, NINJ1, SNAI1, UBTD1, MAP3K6, CDKN2A, E2F1, HDAC4, PLA2R1, PRKCD, WWP1, ID4, NEK1, P3H1, NDRG1, SORBS2) in tumor tissue and normal tissue in the TCGA-THCA cohort were displayed by heat map (Figure 3F). Using consensus clustering analysis based on the expression levels of prognosis-related cellular senescence genes in PTC patients, we investigated the role of those genes in PTC. We divided PTC patients into two clusters (Figure 3G), and details of the consensus cluster analysis are provided in Supplementary Figure 1.

K-M curve shows that cluster 2 had a worse OS (p = 0.021) (Figure 3H), and the clinicopathological heatmap indicated that the number of PTC with pathological type, RAS, BRAF, T-stage, N-stage, M-stage were statistically different between clusters (Figure 3I). These results suggest that these 16 prognostic cellular senescence genes can be used to predict OS in PTC patients and the expression of those genes can provide a new way to differentiate PTC subtypes. The results of the CIBERSORT analysis showed that the content of ten immune cells (T cells CD8, T cells follicular helper, T cells regulatory, Macrophages M0, Macrophages M1, Macrophages M2, Dendritic cells resting, Dendritic cells activated, Mast cells resting, Eosinophils) were statistically different between the two clusters (Figure 3J). Furthermore, the ESTIMATE analysis showed lower ESTIMATEScore, ImmuneScore, StromalScore and higher TumorPurity in cluster 2 (Figure 3K–3N). The results of CIBERSORT and ESTIMATE analysis indicated that cellular senescence genes may affect the prognosis of PTC patients through the immune microenvironment.

To further analyze the reasons for the differing prognosis of the two clusters, we performed KEGG pathway enrichment analysis for clusters 1 and 2, respectively. The results showed that cluster 1 was mainly enriched in allograft rejection, asthma, cell adhesion molecules (CAMs), intestinal immune network for IgA production, leishmania infection, leukocyte transendothelial migration, natural killer cell mediated cytotoxicity, pathogenic *Escherichia coli* infection, type I diabetes mellitus and viral myocarditis. The results showed that cluster 2 was mainly enriched in arginine and proline metabolism, ascorbate and aldarate metabolism, β alanine metabolism, butanoate metabolism, fatty acid metabolism, glycerolipid metabolism, glycosylphosphatidylinositol (GPI)-anchor biosynthesis, lysine degradation, peroxisome, valine leucine and isoleucine degradation. The results of the analysis in cluster 2 suggest that cellular senescence genes may contribute to the worsening of the prognosis of PTC patients through metabolism-related pathways.

### Construction and validation of CSRS

In the training cohort, we constructed CSRS (Supplementary Figure 2) by selecting four genes from 16 prognosis-related cellular senescence genes by LASSO regression analysis for prediction OS. We got the following risk score formula for patients with PTC: Risk score = (0.05013 × expression value of SNAI1) + (0.097623 × expression value of CDKN2A) + (0.54932 × expression value of HDAC4) + (0.02454 × expression value of NDRG1). Based on median the risk score, we divided the patients in the three cohorts into high and low-risk groups.

**Figure 4:**
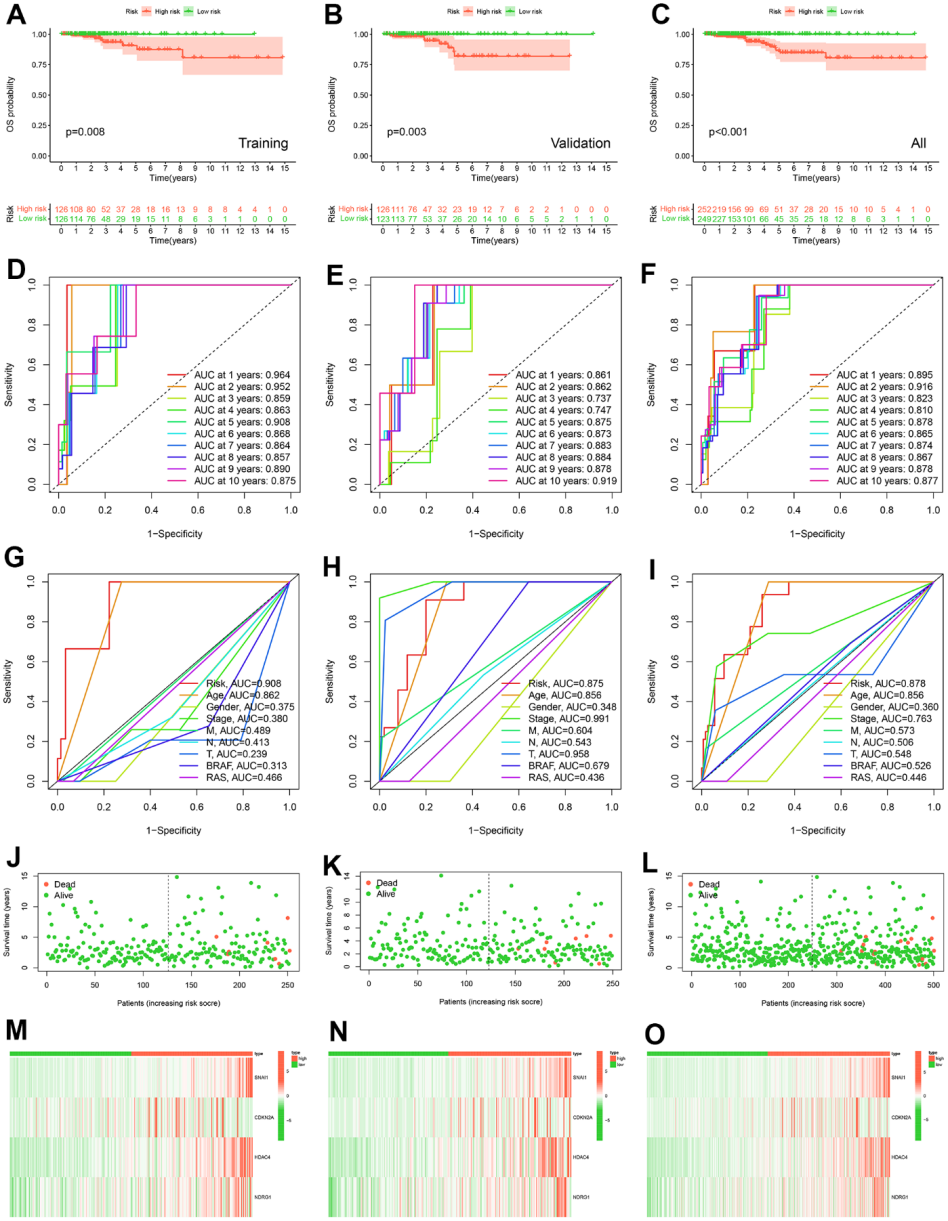
**Construction and validation of prognostic models.** K-M curve of OS probability between high and low risk groups in training cohort (**A**), validation cohort (**B**) and all TCGA-THCA (**C**). The ROC curves of prognostic signature of prognostic model in 1-10 years in training cohort (**D**), in validation cohort (**E**) and in all TCGA-THCA cohort (**F**). The ROC curves of risk score, age, gender, stage, M, N, T, BRAF, and RAS in 5 years in training cohort (**G**), in validation cohort (**H**) and in all TCGA-THCA cohort (**I**). Survival status of patients in the training cohort (**J**), validation cohort (**K**) and all TCGA-THCA (**L**). Expression heatmap of prognostic model genes in the training cohort (**M**), validation cohort (**N**) and all TCGA-THCA (**O**).

**Figure 5:**
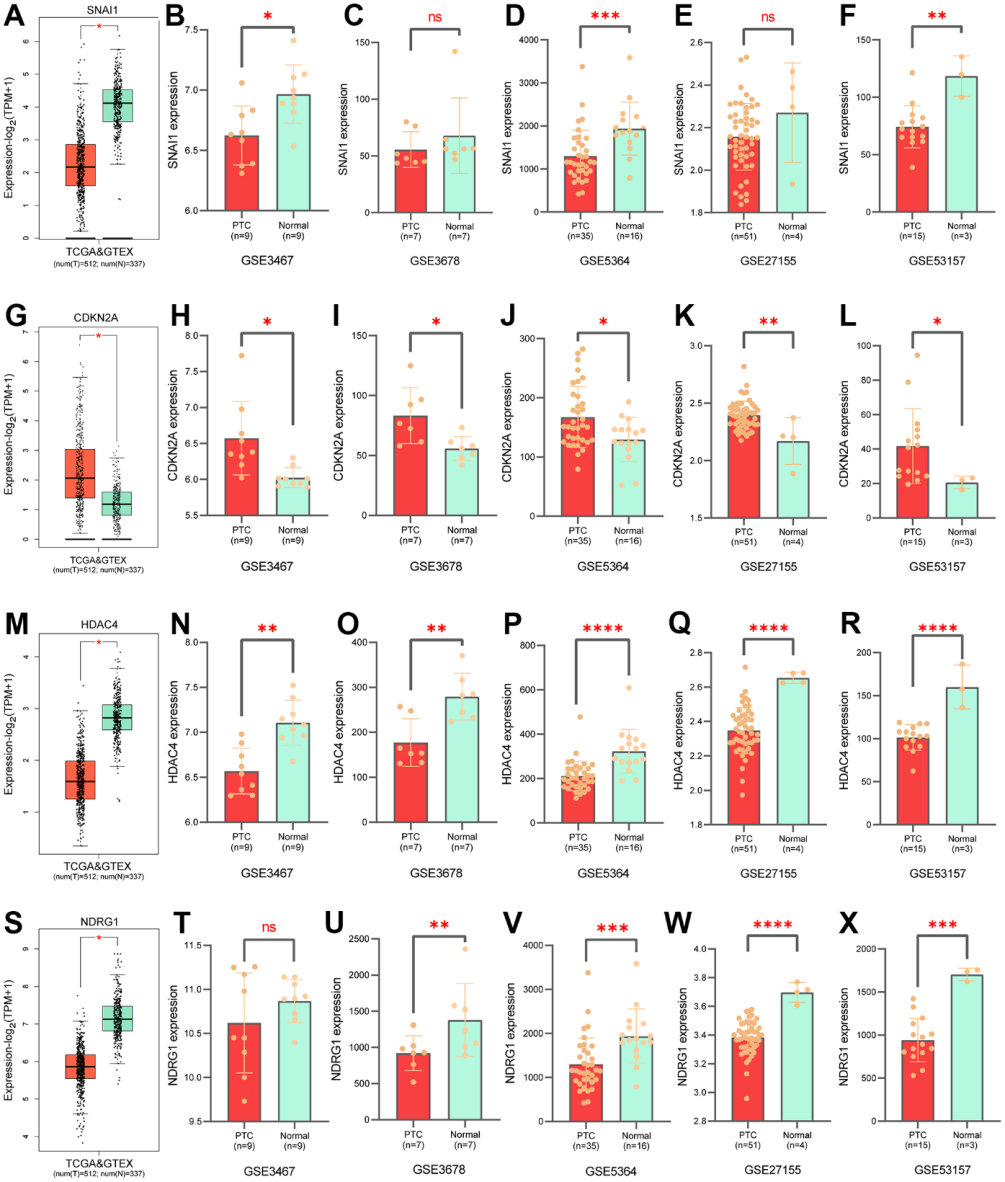
**Validation of gene expression levels in prognostic models.** Expression of SNAI1 in GTEx (**A**), in GSE3467 (**B**), in GSE3678 (**C**), in GSE5364 (**D**), in GSE27155 (**E**), in GSE35157 (**F**). Expression of CDKN2A in GTEx (**G**), in GSE3467 (**H**), in GSE3678 (**I**), in GSE5364 (**J**), in GSE27155 (**K**), in GSE35157 (**L**). Expression of HDAC4 in GTEx (**M**), in GSE3467 (**N**), in GSE3678 (**O**), in GSE5364 (**P**), in GSE27155 (**Q**), in GSE35157 (**R**). Expression of NDRG1 in GTEx (**S**), in GSE3467 (**T**), in GSE3678 (**U**), in GSE5364 (**V**), in GSE27155 (**W**), in GSE35157 (**X**).

The K-M survival curve analysis showed that patients with high-risk groups had shorter OS (Figure 4A–4C). The AUC value of PTC patients in 1-10 years was greater than 0.737 that the model has good accuracy (Figure 4D–4F). To compare the predictive power of CSRS and clinicopathological characteristics for OS, we evaluated the AUC value of CSRS and clinical pathological characteristics in the fifth year. The results demonstrated that the risk scores achieved the highest AUC value than other clinical pathological characteristics (Figure 4G–4I). These results indicated that CSRS has good predictive ability and clinical application value. Survival status, a heatmap of 4-gene expression levels, distribution of risk scores in high and low risk groups (Figure 4J–4O and Supplementary Figure 3). We analyzed gene expression data from the GEPIA2 website and five GEO cohorts, and the results further supported the previous conclusion (Figure 5) (the SNAI1 gene expression level showed a trend of lower expression in tumor tissues in GSE3678 and GSE27155, Figure 5A–5X).

### The risk score in hierarchical analysis is the predictor for PTC

**Figure 6:**
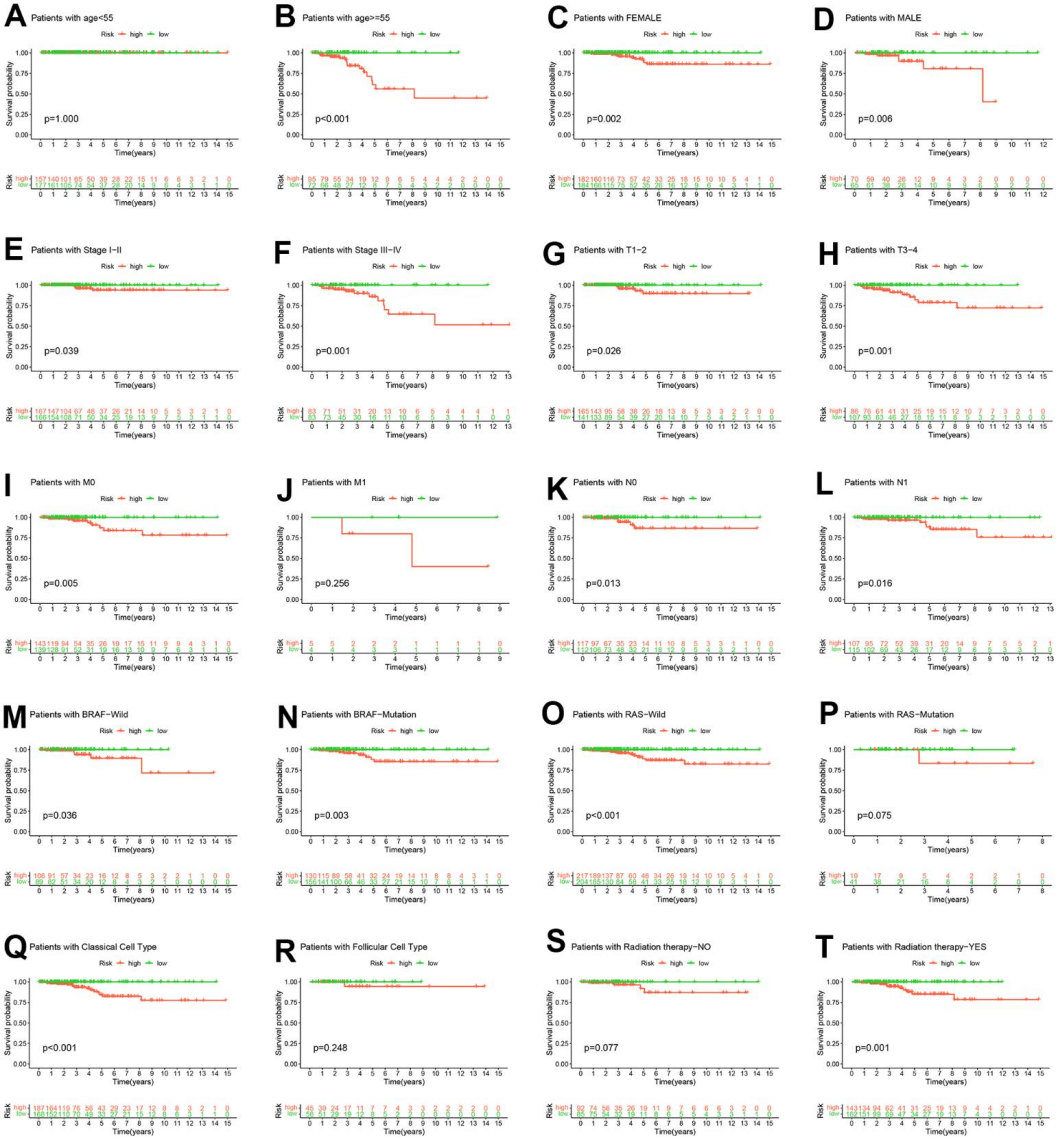
**Hierarchical analysis.** K-M curve analysis of OS probability was based on risk scores grouped by age (**A**, **B**), sex (**C**, **D**), stage (**E**, **F**), T (**G**, **H**), M (**I**, **J**), N (**K**, **L**), BRAF (**M**, **N**), RAS (**O**, **P**), pathological subtype (**Q**, **R**) and radiation therapy (**S**, **T**).

To further mitigate the effect of clinicopathological features on risk scores predicting OS, we conducted a stratified analysis based on the clinicopathological features of the entire TCGA-THCA cohort. The K-M survival curve results in the stratified analysis showed that the high-risk group had a shorter OS, except for patients with age < 55, M1, RAS-Mutation, Follicular Cell Type, Radiation therapy−NO (Figure 6).

### Multiple omics differences between high - and low-risk groups

**Figure 7:**
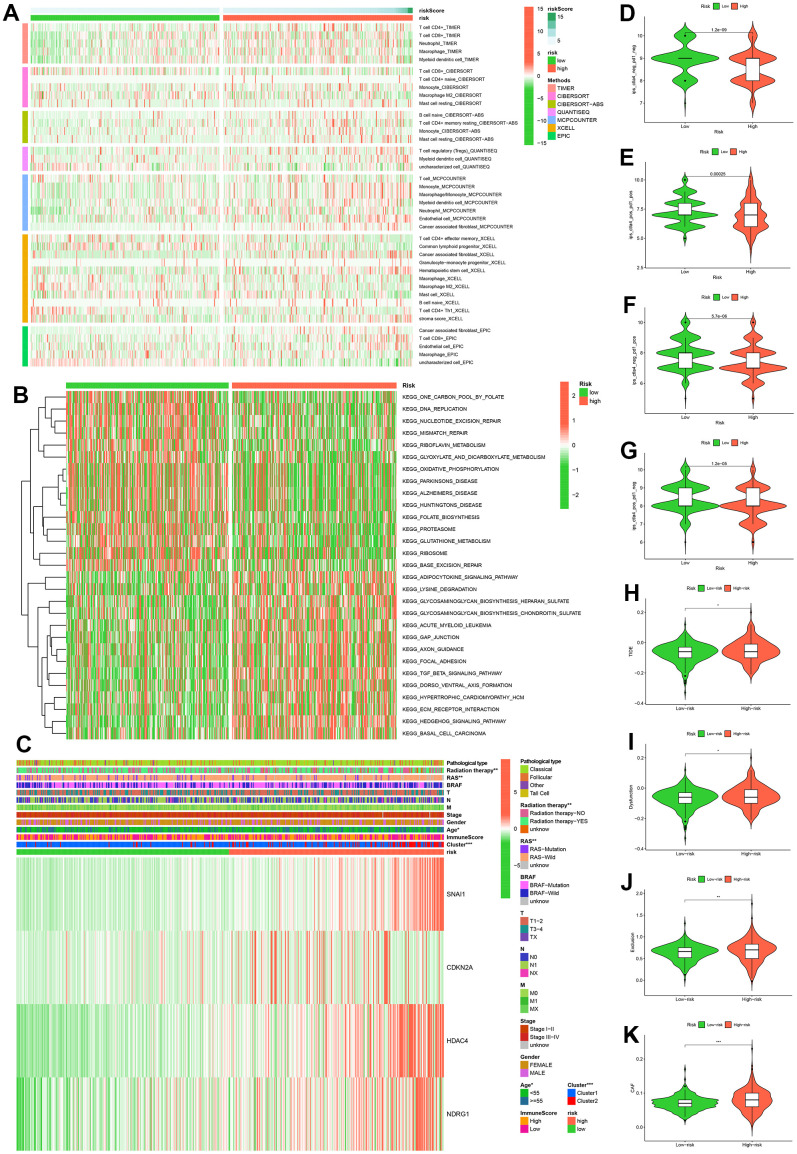
**Multi-omics analysis.** Analysis results of six immune cell infiltration algorithms in high and low risk groups (**A**). KEGG pathway enrichment analysis in high and low risk groups (**B**). Heatmap of clinicopathological features in high and low risk groups (**C**). Comparison of immunophenoscores (IPS) between high and low risk groups who have not been treated with anti-CTLA4 or anti-PD-1 immunotherapy (**D**), who have been treated with anti-CTLA4 and anti-PD-1 immunotherapy (**E**), who have been treated with anti-CTLA4 and anti-PD-1 immunotherapy, who have only been treated with anti-PD-1 immunotherapy (**F**), and who have only been treated with anti-CTLA4 immunotherapy (**G**). Comparison of TIDE (**H**), Dysfunction (**I**), Exclusion (**J**), CAF (**K**) between high and low risk groups.

A heatmap of immune cells with statistically significant differences between high and low risk groups based on the analysis algorithms of immune cell contents (TIMER, CIBERSORT, CIBERSORT-ABS, QUANTISEQ, MCPCOUNTER, XCELL, EPIC) was presented in Figure 7A. These results provide a basis for further elucidating the relationship between cell senescence and immune cells. To investigate the biological behavior of the high-risk group, we analyzed by GSVA and found that the high-risk group was mainly enriched pathway in adipocytokine signaling pathway, lysine degradation, glycosaminoglycan biosynthesis-heparan sulfate, glycosaminoglycan biosynthesis-chondroitin sulfate, acute myeloid leukemia, gap junction, axon guidance, focal adhesion, TGF-β signaling pathway, dorso-ventral axis formation, hypertrophic cardiomyopathy (HCM), ECM-receptor interaction, hedgehog signaling pathway, basal cell carcinoma (Figure 7B). Furthermore, the heat map of the correlation between clinicopathological characteristics and risk scores showed that there were statistical differences in radiation therapy, RAS, Age and Cluster in the high and low risk groups (Figure 7C). Additionally, four IPS scores were statistically different between high and low risk groups (Figure 7D–7G). The data provided by the TIDE website showed that TIDE scores, Dysfunction scores, Exclusion scores and CAF scores were statistically different between high and low risk groups (Figure 7H–7K).

**Figure 8:**
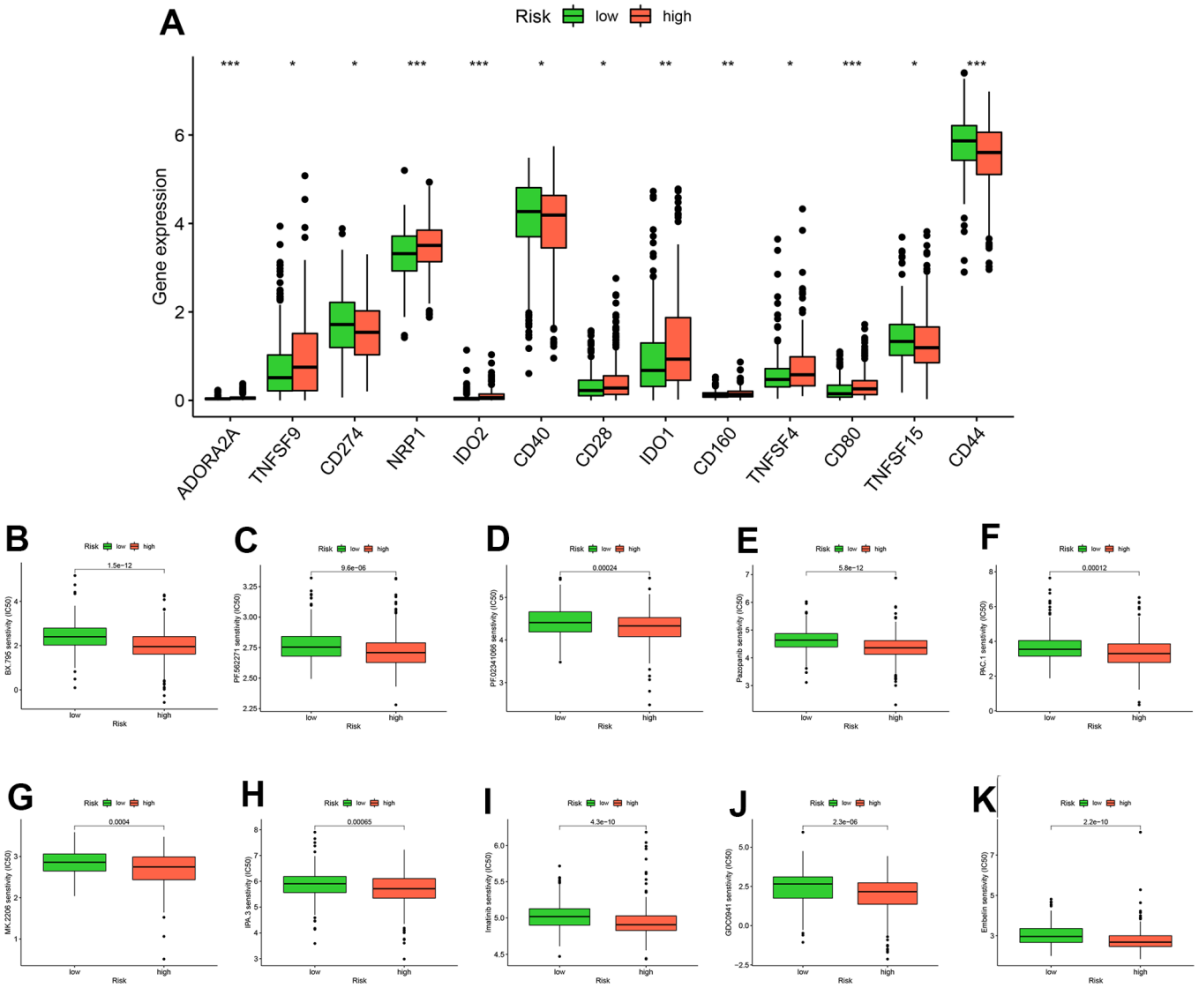
**Analysis of drug treatment.** There are differences in immune checkpoint expression between groups at high and low risk (**A**). Ten drugs with lower half-maximum inhibitory concentration in the high-risk group (**B**–**K**).

Through an analyzing the expression levels of immune checkpoints, we found that 13 immune checkpoints (ADORA2A, TNFSF9, CD274, NRP1, IDO2, CD40, CD28, IDO1, CD160, TNFSF4, CD80, TNFSF15, CD44) were differentially expressed in high and low risk groups (Figure 7A). Subsequently, we identified ten drugs (BX.795, PF.562271, PF.02341066, Pazopanib, PAC.1, MK.2206, IPA.3, Imatinib, GDC0941, Embelin) that were more sensitive in high-risk patients by drug sensitivity analysis (Figure 8B–8K).

## DISCUSSION

Aging is a significant contributing factor to death and illness, especially cancer [12] and is closely associated with cellular senescence. Two studies, utilizing data from The Cancer Genome Atlas (TCGA), have identified robust correlations between a patient’s age and various aspects of pan-cancer, including the genome, transcriptome, epigenetics, copy number alterations, structural rearrangements, immune cell infiltration, and immune checkpoints [47, 48]. Shah et al. further emphasized specific differences in signal among different tumor types through multi-omics analyses, and pointed out that TC had the highest HR values when HR for OS were used as an indicator of age at tumor diagnosis [12]. We obtained similar results by analyzing SEER cohort and TCGA-THCA cohort. Another study showed that upregulated genes in TC significantly overlap with overexpressed senescence genes, and were the only cancer type with this condition and cellular senescence was associated with younger TC patients [49]. Studies have also revealed the presence of senescent tumor cells in the leading edge of collective invasion in PTC, as well as lymphatic and lymph node metastases, and senescence-associated secretory phenotype (SASP) of aging tumor cells exhibit high invasive capacity [50, 51].

Several studies have highlighted the potential of cellular senescence-related genes can be used as prognostic indicators of tumors [52, 53]. This study screened 76 cellular senescence genes by TCGA-THCA cohort and eight GEO cohorts for differential expression between tumor and normal tissues. GO and KEGG enrichment analyses showed that 76 genes were closely related to aging and p53 signaling pathways. Further PPI network showed that P53 protein had more interactions with other proteins. These results are consistent with previous conclusions that P53 is considered to be a hallmark of cellular senescence [54]. We further performed univariate Cox analysis to investigate the relationship between the expression levels of 76 genes and the OS of PTC patients to find 16 prognosis-related cellular senescence genes. Consensus clustering analysis was performed on PTC patients according to the expression levels of these 16 genes and the patients were divided into two clusters. Among them, cluster 2 had a worse OS. These results suggest that cellular senescence genes can be used to predict OS in PTC patients. Through further LASSO regression analysis, we screened out four genes (SNAI1, CDKN2A, HDAC4, NDRG1) to construct a prognostic model.

Each gene within the signature underwent a detailed analysis to gain a better understanding of its functions. SNAI1 is a zinc finger transcription factor that induces epithelial-mesenchymal transition (EMT) in various cancers and epithelial cells [55]. Previous studies have shown that downregulating SNAI1 expression can suppress EMT and slow down the progression of PTC [56–58]. It has been reported that inhibition of SNAI1 can induce cellular senescence in prostate cancer and various cell lines, but the relationship between SNAI1 and senescence in PTC remains unclear [55, 59]. The CDKN2A (also known as ARF, INK4A, MTS-1) encodes two tumor suppressor proteins p16INK4A and p14ARF [60]. Previous study reported that high p16INK4A expression in PTC was associated with poor prognosis [61], and our findings suggested that CDKN2A was a risk factor for PTC patients. Based on a meta-analysis of 734 PTC patients, it was found that cancer tissues had significantly more CDKN2A promoter methylation than benign and normal tissues [62]. The p16INK4A is considered a marker of cellular senescence in a variety of diseases, including PTC, but the specific role of the p16INK4A is unclear [50, 51, 63]. Class IIa histone deacetylase 4 (HDAC4), a zinc-dependent enzyme, deacetylates histones by removing acetyl groups in a zinc-containing catalytic domain, resulting in the condensation of nucleosomes in the nucleus [64]. HDAC4 is polyubiquitinated and degraded during all types of senescence [65] and our findings also suggest that the mRNA expression of HDAC4 is decreased in PTC tissues. Previous evidence has revealed that triple mutants of HDAC4 in fibroblasts can trigger TP53 stabilization and oncogene-induced senescence, and HDAC4 inhibits p16INK4A promoter activity in a dose-dependent manner [65, 66]. NDRG1 belongs to the NDRG protein family and has been extensively demonstrated to possess anti-oncogenic and anti-metastatic properties [67]. A study showed that the inhibition of NDRG1 suppresses hepatocellular carcinoma growth and reactivates senescence signaling [67]. However, the relationship of NDRG1 in PTC to cellular senescence requires further exploration.

The K-M survival curve and ROC curve results for the training dataset, validation dataset and the whole TCGA-THCA showed that the CSRS had a good ability to predict the OS of PTC. Even after accounting for the effect of clinicopathological features on the prognosis of patients with PTC, patients with high-risk scores had poorer OS. The results of previous studies have demonstrated that when PTC occurs and develops, the immune system level increases, and the proportion of tumor-promoting immune cells increases substantially [68]. In our investigation, we applied seven different algorithms to analyze immune cell populations that differed between high and low-risk groups. Furthermore, we conducted an analysis of KEGG pathways enriched in the high and low-risk groups. Notably, distinct results were observed in terms of immune cell populations and enriched KEGG pathways between the high and low-risk groups. Thus, elucidating the role of cellular senescence in PTC through the lens of immune cells and KEGG pathway analysis represents a promising avenue for future research. Moreover, IPS serves as an evaluative index for ICI treatments, and higher IPS means better immunotherapy results. Utilizing the TIDE algorithm, we predicted the ICI response rates for two patient subtypes and assessed whether the risk score might benefit PTC patients undergoing immunotherapy. Our study revealed statistically significant differences in IPS and TIDE scores between patients with high risk and those with low risk, suggesting that immunotherapy may be advantageous for high-risk patients. Further immune checkpoint and drug sensitivity analysis identified 13 potential immune checkpoints (ADORA2A, TNFSF9, CD274, NRP1, IDO2, CD40, CD28, IDO1, CD160, TNFSF4, CD80, TNFSF15, CD44) and ten sensitive drugs (BX.795, PF.562271, PF.02341066, Pazopanib, PAC.1, MK.2206, IPA.3, Imatinib, GDC0941, Embelin). This provides potential therapeutic targets and drugs for inducing cell senescence in PTC.

Our study had some limitations. Firstly, our consensus cluster analysis and CSRS were solely based on TCGA-THCA, without multi-center clinical information and sequencing data for verification, which may weaken the clinical promotion value of CSRS. Finally, the specific mechanism of action of the four key genes screened in the prognostic model has not been explored *in vitro* and *in vivo*, which requires further studies in PTC.

## CONCLUSIONS

Consequently, our study has identified and validated CSRS as a prognostic significance marker for patients with PTC based on four genes related to cellular senescence. Finally, we have demonstrated an intricate relationship between these risk scores and their implications for immunotherapy, drug therapy, and immune checkpoint genes. The combination of risk scores with specific immune checkpoint factors could be used to develop predictive biomarkers of ICI response. This approach may enable a more precise selection of patients who are likely to benefit from checkpoint inhibitors. As a consequence, identifying and further researching cellular senescence-related genes involved in tumor immune response might assist with the risk stratification of PTC and PTC may be treated more effectively with immunotherapy if these targets are identified.

## Supplementary Material

Supplementary Figures

Supplementary Table 1

Supplementary Table 2

Supplementary Table 3
