# Supplementary material for: Comprehensive analysis of cellular senescence and immune microenvironment in papillary thyroid carcinoma
Source: Aging (Albany NY). 2024 Feb 7;16(3):2866–86. doi: 10.18632/aging.205520 (PMC10911381; doi:10.18632/aging.205520)
Supplement: Supplementary Figures [file aging-16-205520-s001.pdf]

## SUPPLEMENTARY FIGURES

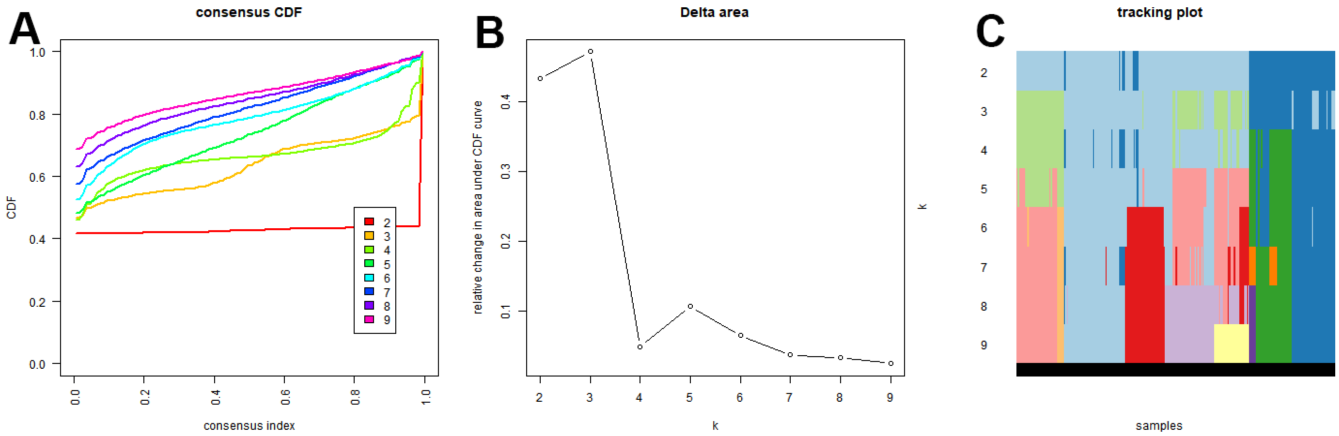


**Supplementary Figure 1. Consensus cluster analysis process.** Results of consensus cumulative distribution function analysis (**A**), Delta area (**B**) and Tracking plot (**C**).

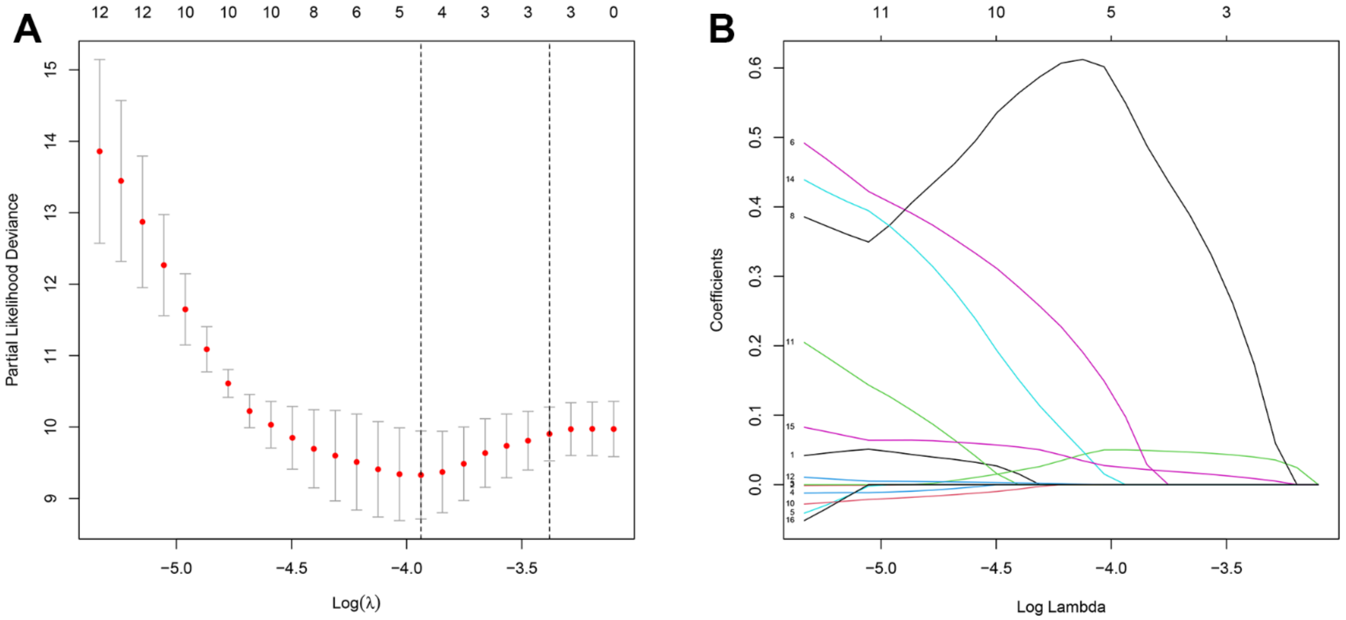


**Supplementary Figure 2. Determination of prognostic model.** (**A**) LASSO coefficient path map for 16 risk factors; (**B**) Cross verification curve.

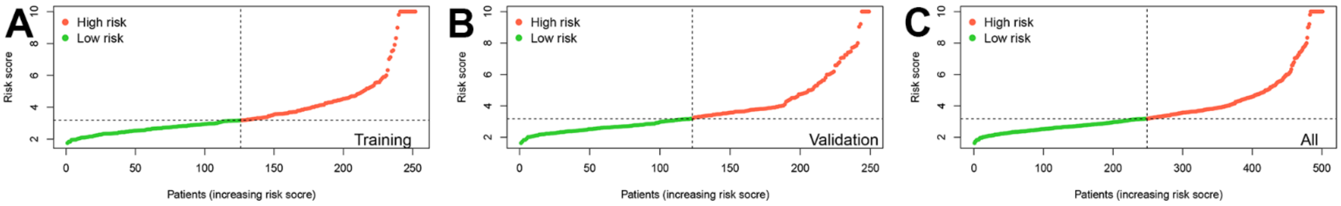


**Supplementary Figure 3. Distribution of risk scores in the training cohort, validation cohort, and all TCGA-THCA cohort.** Rank the patient's risk scores in the training cohort (**A**), validation cohort (**B**) and entire cohort (**C**).
